# Circulating Tumor DNA Is Capable of Monitoring the Therapeutic Response and Resistance in Advanced Colorectal Cancer Patients Undergoing Combined Target and Chemotherapy

**DOI:** 10.3389/fonc.2020.00466

**Published:** 2020-04-07

**Authors:** Hua Cao, Xinyi Liu, Yixin Chen, Pan Yang, Tanxiao Huang, Lele Song, Ruilian Xu

**Affiliations:** ^1^Department of Oncology, Shenzhen People's Hospital, The 2^nd^ Clinical Medical School of Ji'nan University, The First Affiliated Hospital of Southern University of Science and Technology, Shenzhen, China; ^2^HaploX Biotechnology, Co., Ltd., Shenzhen, China

**Keywords:** colorectal cancer, ctDNA, NGS, sequencing, bevacizumab, cetuximab, monitoring, resistance

## Abstract

Colorectal cancer (CRC) is a highly lethal disease worldwide. The majority of patients receiving targeted therapy or chemotherapy develop drug resistance, while its molecular mechanism remains to be elucidated. The plasma circulating tumor DNA (ctDNA) exhibited the potential in identifying gene variations and monitoring drug resistance in CRC treatment. In this study, we monitored the ctDNA mutational changes in advanced CRC patients underwent first-line therapy with bevacizumab and cetuximab combined with chemotherapy. The mutation spectrum of 43 patients was established by a 605-gene next-generation sequencing (NGS) panel. The baseline measurement shows that genes with the highest mutation frequency were TP53 (74%), APC (58%), KRAS (40%), SYNE1 (33%), LRP1B (23%), TOP1 (23%), and PIK3CA (21%). Mutations in TP53, APC, and KRAS were detected in 29 paired plasma and tissue samples with the consistency of 81, 67, and 42%, respectively. Clinically targetable gene mutations, such as APC, RNF43, SMAD4, BRAD1, KRAS, RAF1, and TP53, were also identified in ctDNA. The overall consistency between ctDNA and tissue samples was 54.6%. Alleviation of mutational burden in BRAF, KRAS, AMER1, and other major driving genes was observed following the first-line therapy. Patients with KRAS and TP53 mutations in tissues appeared to benefit more than the wild-type counterpart. The dynamic change of plasma mutation status was consistent with the tissue tumor burden and was closely correlated with disease progression. In conclusion, ctDNA monitoring is a useful method for molecular genotyping of colorectal cancer patients. Dynamic changes in resistance can be sensitively monitored by gene variation status, which potentially helps to develop treatment strategy.

## Introduction

Colorectal cancer is one of the most common malignant tumors worldwide, responsible for the second and fourth highest mortality among men and women, respectively (1, 2). Standard therapies, including chemotherapy, radiotherapy, targeted therapy, and surgery, are frequently used for colorectal cancer treatment. However, tumor cells can evolve under the pressure of treatment selectivity. Therefore, elucidating the genetic alterations that may potentially drive tumor cell resilience is crucial to advance the knowledge in cancer therapy (2).

Liquid biopsy has been widely recognized as a real time monitoring method to detect tumor-related genetic alterations (3). In fact, liquid biopsy has been largely used to analyze circulating tumor DNA (ctDNA), circulating tumor cells (CTC) and exosomes isolated from peripheral blood. This method can potentially recognize, at the genomic level, tumors associated with lower invasiveness (4). CTC, exosomes, and ctDNAs are widely used in clinical diagnosis and in treatment monitoring, however, there are still some limitations that deserve improvement. Among these three types of biological materials, ctDNA has unique advantage in monitoring the tumor genotype with the development of next generation sequencing (NGS). It is recognized as a method to reveal tumor genome information and can be used to discover cancer evolution and clonal heterogeneity. Therefore, it can be used as a tumor biomarker to evaluate treatment efficiency and drug resistance (5–9).

The NGS technology has been used to monitor changes on ctDNA levels in peripheral blood and in the dynamic change of drug-resistant genes, therefore allowing the selection of novel therapeutic approaches and drug strategies. Due to its effectiveness and fast accessibility, NGS has been widely used in liquid biopsies to analyze genomic alterations in the peripheral blood of cancer patients. In the present study, we used an established sequencing workflow to detect genomic alterations in tumor tissues and peripheral blood from CRC patients who have received first-line therapy with bevacizumab and cetuximab combined with chemotherapy. The NGS panel containing 605 tumor-associated genes was used to detect the main driver gene mutations, resistance-related mutations, and to track dynamic changes of tumor ctDNA during treatment. Our observation suggests that it is vital to identify CRC patients with drug resistance during treatment and adapt the treatment strategy accordingly.

## Methods and Materials

### Patients and Samples

A prospective cohort study was designed and implemented in the Shenzhen People’s Hospital (Shenzhen, China). Blood samples and intestinal tumor tissues were collected at the Shenzhen People’s Hospital. This research was approved by the Shenzhen People’s Hospital Ethics Committee and conducted in accordance with its guiding principles. All patients received written informed consent for the use of clinical samples. Patient information was kept anonymous for confidentiality. The main inclusion criteria include adults over 18 years old and those have complete clinicopathological information and confirmed diagnosis of CRC by imaging examination (including endoscopy, ultrasound, MRI, CT, etc.) and/or subsequent pathological examination. Subjects were included for those who have the indications for adjuvant chemotherapy, neoadjuvant chemotherapy, or chemotherapy combined with target therapy (target therapy includes but is not limited to cetuximab, apatinib, bevacizumab, and trastuzumab) based on current condition. Patients were also included for those with blood samples and/or tissue samples available before the start of the current therapy, and those who can be followed up and agree to provide the subsequent blood samples during and after therapy. The main exclusion criteria include pregnant woman, and those who have history of cancers other than CRC, or history of therapy on other cancers. Subjects were excluded for those with no blood and/or tissue samples available before the start of therapy or those not available for follow-up or cannot provide blood samples during and after therapy. Patients with incomplete information were also excluded. As a result, a cohort of 41 patients with advanced CRC (excluding two patients with stage I CRC), treated with cetuximab, apatinib, bevacizumab, trastuzumab, neoadjuvant, adjuvant chemotherapy, or any combination, was enrolled into the study. Tissue samples were prepared from formalin-fixed and paraffin-embedded (FFPE) samples and 10 ml peripheral blood samples were collected with anticoagulant tubes. The patient clinical information related to each sample is shown in Table 1. Progression free survival (PFS) was used to assess the effectiveness of therapy.

**Table 1:** Demographic and clinical characteristics of study participants.

**Characteristics**	**Patients**
**Age, year, median (range)**	43 (25–84)
**Sex**, ***n*** **(%)**	
Male	31 (72.1%)
Female	12 (27.9%)
**Anatomical position of primary lesion**, ***n*** **(%)**	
Transverse colon	1 (2.33%)
Cecum	1 (2.33%)
Sigmoid colon	10 (23.26%)
Left colon	5 (11.63%)
Right colon	9 (20.93%)
Rectum	15 (34.88%)
The junction of rectum and sigmoid colon	2 (4.65%)
**Stage**, ***n*** **(%)**	
1	2 (4.65%)
3A	2 (4.65%)
3B	3 (6.98%)
3C	1 (2.33%)
4	35 (81.4%)
**Resection of primary tumor**, ***n*** **(%)**	
Yes	27 (62.79%)
No	16 (37.21%)
**Metastatic site**, ***n*** **(%)**	
Lung	6 (13.95%)
Liver	11 (25.58%)
Lymph gland	7 (16.28%)
Peritoneum	3 (6.98%)
Multi-organ	8 (18.6%)
Others	2 (4.65%)
None	6 (13.95%)
**Treatment line at the time of baseline sampling**, ***n*** **(%)**	
1st line	5 (11.63%)
2nd line	2 (4.65%)
3rd line	2 (4.65%)
Adjuvant chemotherapy	2 (4.65%)
Neoadjuvant chemotherapy	8 (18.6%)
None	24 (55.81%)
**Baseline sample**, ***n*** **(%)**	
Blood	4 (9.3%)
Tissue	20 (46.51%)
Tissue+blood	19 (44.19%)
**Number of blood samples at monitoring points**, ***n*** **(%)**	
0	24 (55.81%)
1	12 (27.91%)
2	6 (13.95%)
4	1 (2.33%)
**RAS status in tissue**, ***n*** **(%)**	
Wild	26 (60.47%)
Mut	17 (39.53%)

### DNA Extraction and Quantification

For the FFPE samples, ten 5 μm tumor slices were used for DNA extraction using the QIAamp DNA FFPE Kit (QIAGEN, Valencia, CA, USA) following the manufacturer's instructions. DNA from fresh tissue samples was extracted using the EasyPure^®^ Genomic DNA Kit (Beijing TransGen Biotech, Beijing, China). Blood samples from patients were collected in Ethylene Diamine Tetraacetic Acid (EDTA) tubes and centrifuged at 1,600 g for 10 min and at 4°C. The supernatants were further centrifuged at 10,000 × g for 10 min at 4°C, and plasma was harvested and stored at −80°C until further use. ctDNA was extracted from 3 to 3.5 ml plasma using the QIAamp Circulating Nucleic Acid kit (Qiagen, Inc., Valencia, CA, USA) according to the manufacturers' instructions. Blood cell fragments (including peripheral blood lymphocytes and red cells) were preserved at −20°C for further study. We applied the RelaxGene blood DNA system (Tiangen Biotech) to extract genomic DNA from peripheral blood lymphocytes (PBLs) as the normal control for mutation calling from cancer tissues and ctDNA. The quality control of the DNA was achieved using Qubit 2.0 (Thermo Fisher Scientific), in accordance with manufacturer's instructions.

### Library Construction and Sequencing

DNA from blood samples was cleaved using a double-stranded DNA Fragmentase (Roche Sequencing and Life Science, Indianapolis, IL 46250, USA). The construction of the ctDNA library was performed using a KAPA Library preparation kit (KAPA Biosystems, Wilmington, MA 01887, USA). Sequencing was performed to an average depth of 5,000 × on Illumina Novaseq6000. A sequencing panel of 605 genes targeting the exome regions was used to identify mutations. The logarithmic ratio of each gene region was properly computed. WESPlus gene panel (an upgraded version of the standard whole-exome sequencing (WES) (HaploX Biotechnology) for cancer tissue sequencing. Seven to eight polymerase chain reaction (PCR) cycles, depending on the amount of DNA input, were performed on Pre-LM-PCR Oligos (Kapa Biosystems, Inc.) in 50 μl reactions. DNA sequencing was then performed on the Illumina Novaseq 6000 system according to the manufacturer's instructions. Data which meet the following criteria were chosen for subsequent analysis: the ratio of remaining data filtered by fastq in raw data is ≥85%; the proportion of Q30 bases is ≥85%; the ratio of reads on the reference genome is ≥85%; target region coverage ≥98%; average sequencing depth in tissues is ≥500×; average sequencing depth in blood cfDNA is ≥1,500 #x000D7;. The called somatic variants need to meet the following criteria: the read depth at a position is ≥20×; the variant allele frequency (VAF) is ≥2% for tissue DNA and ≥0.05% for cfDNA from blood; somatic-*P* ≤ 0.01; strand filter ≥1. Allele frequencies were calculated for Q30 bases. The copy number variation was detected by CNVkit version 0.9.3 (https://github.com/etal/cnvkit). Further analyses of genomic alterations were also performed, including single nucleotide variants (SNVs), copy number variations (CNVs), insertion/deletion (Indels), fusions, and structural variation. Tumor mutation burden (TMB) was referred as the total number of incorrect coding, base substitution, insertion, and deletion in somatic cells per million bases.

### Statistical Analysis

**Figure 1:**
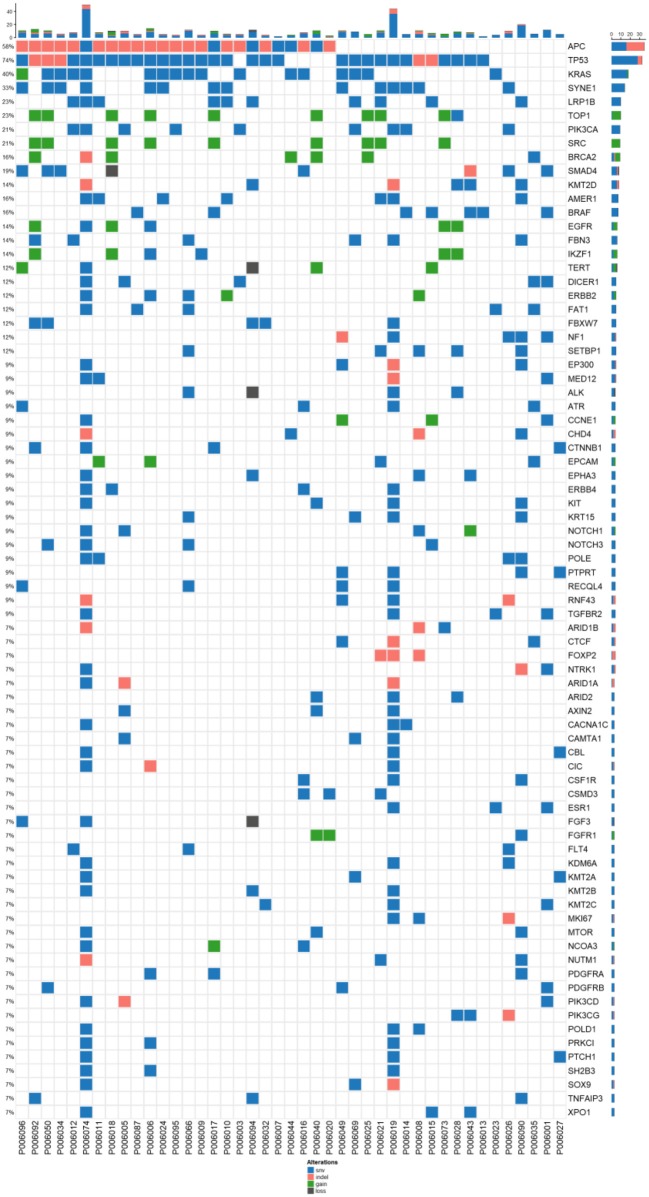
Mutation spectrum of baseline plasma and tissue samples from 43 patients CRC. The genes with a high mutation frequency among all patients are listed on the left and individual patients are represented by the columns. Mutation types of non-synonymous single nucleotide variant, SNV (single nucleotide variant), indel (insertion-deletion), gain(stop gain, non-sense mutation), and loss(stop loss, missense mutation) are represented by blue, orange, and dark, respectively.

**Figure 2:**
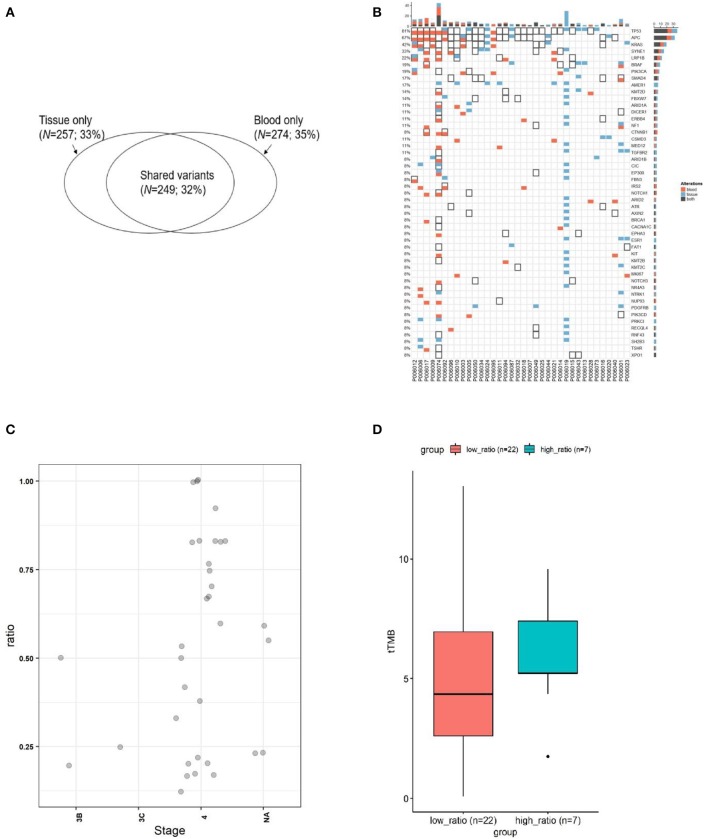
The concordance of mutation between ctDNA and the corresponding tumor tissues from CRC. **(A)** Wenn diagram shows the number and percentage of overlapped and unique mutations between the tissue DNA and the corresponding blood ctDNA samples. **(B)** Mutation spectrum of baseline plasma and the corresponding tumor tissues from 35 subjects. **(C)** The mutation concordance ratio for different stages between blood ctDNA samples and tumor tissue samples from 28 patients. **(D)** Comparison of tumor mutation burden (TMB) in low consistency (<75%) and high consistency (>75%) groups.

**Figure 3:**
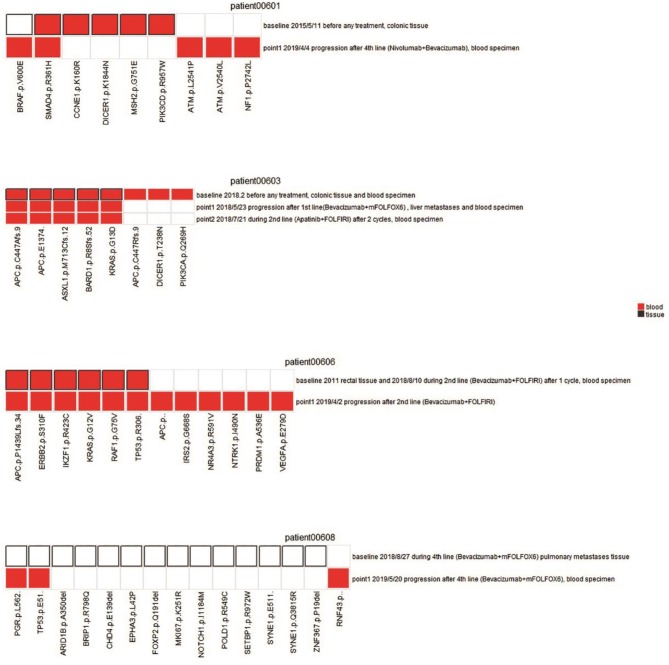
Comparison of mutation status between plasma and tissues in four patients. Mutations for both pre-therapeutic tissue or blood samples and post-therapeutic blood samples were compared. Red squares indicate mutations from blood, and white squares with black solid border indicate mutations from tissue. Red squares with black solid border indicate mutations found in both blood and tissue. White squares with light gray border indicate no mutations.

**Figure 4:**
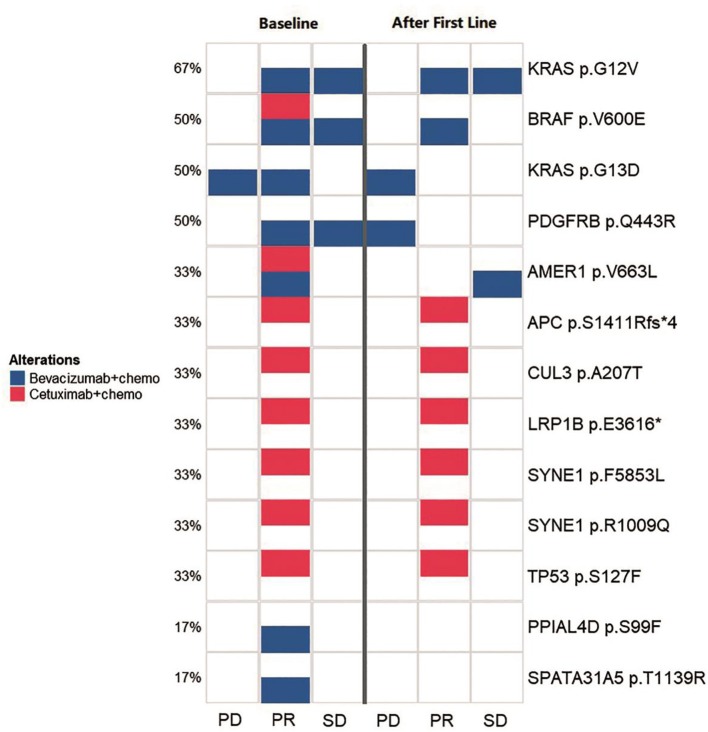
Comparison of the spectrum of key mutations before and after the first-line therapy. The left three lanes (baseline group) and the right three lanes (after first line group) indicate the high-frequency mutational status for baseline (before the first-line therapy) and after the first-line therapy. Annotations to the right of the panel show the exact mutations and the percentage to the left of the panel shows the frequency for a certain mutation. The three lanes in both group illustrates the mutational status for PD, PR, and SD groups, respectively. Blue squares indicates cases with bevacizumab/chemotherapy that carried corresponding labeled mutations and red squares indicates cases with cetuximab/chemotherapy that carried corresponding labeled mutations.

**Figure 5:**
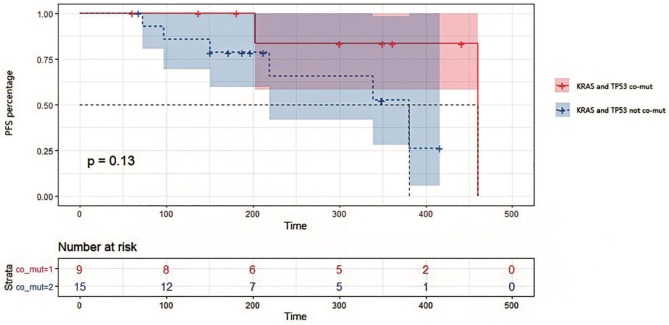
Kaplan-Meier survival analysis on progression-free survival in patients with or without KRAS and TP53 co-mutations in tissues. Survival curves were compared by Log-rank (Mantel-Cox) test. *P* = 0.13 for statistical comparison between the two groups. Data was represented with 95% confidence interval.

All charts and data analyses were performed using R statistical software package (https://www.r-project.org/). The significant difference of TMB in tumor tissues was determined by Student's *t*-test. According to the type of *KRAS* and *TP53* mutation identified (i.e., common or non-common). Survival curves were compared by Log-rank (Mantel-Cox) test. *P* < 0.05 was considered statistically significant. Data was represented with 95% confidence interval. Several packages of the R software were used to plot some figures, including the “ComplexHeatmaps” package (Figures 1, 2B, 3, 4), the “ggplot2” package (Figures 2C,D), and the “survival” package (Figure 5).

## Results

### Mutation Profiling of CRC Patients Before Therapy in Tissue and Blood

In order to verify the feasibility of ctDNA in peripheral blood by NGS, we first recruited 43 CRC patients (including 41 stage III-IV and 2 stage I patients) undergoing chemotherapy combined with target therapy agents (cetuximab, apatinib, trastuzumab) in neoadjuvant and/or adjuvant therapy. The baseline clinical characteristics of these patients are shown in Table 1. Their median age was 53 years old (ranged from 25 to 84 years old). The majority of the CRC patients were male (*n* = 31, 72.1%). The most frequent site of metastasis was the liver (*n* = 11, 25.58%), followed by lymph node (*n* = 7, 16.28%), lungs (*n* = 6, 13.95%), and peritoneum (*n* = 3, 6.98%). Among all patients, a total of 26 (60.47%) presented wild-type RAS. The number of patients with stage I, III, and IV was 2 (4.65%), 6 (13.96%), and 35 (81.4%), respectively. Two patients were treated with adjuvant chemotherapy (4.65%), while 8 were treated with neoadjuvant chemotherapy (18.6%), 5 patients were at the first-line therapy (11.63%), and 2 patients at the second-line therapy (4.65%), and 2 patients at the third-line therapy (4.65%). A total of 24 patients were solely treated with chemotherapy (55.81%).

Among 41 advanced colorectal cancers, 78 genes with high frequency mutations were identified. The top ten highly mutated genes were *TP53* (74%), *APC* (58%), *KRAS* (40%), *SYNE1* (33%), *LRP1B* (23%), *TOP1* (23%), *PIK3CA* (21%), *SRC* (21%), *BRCA2* (16%), and *SMAD4* (19%) (Figure 1). The most frequent SNV mutations were observed in *KRAS* (*n* = 32, 74.4%), *TP53* (*n* = 18, 41.8%), and *SYNE1* (*n* = 23, 53.5%). The most frequent Indel mutations were observed in APC3 gene in 25 of 43 patients (58.1%). The most frequent CNVs occurred in *TOP1* and *SRC* (Figure 1).

Blood and tissue samples were collected from all patients before receiving any therapy. By comparing the mutational rates (and their consistency) in tissues and plasma, we found that 249 out of 506 mutations detected in tissue samples were also found in the corresponding ctDNA samples (Figure 2A). The consistency calculated on mutational sites between tissue and blood samples was 32%. Our sequencing strategy enabled the detection of SNVs, indels, CNVs, and gene fusions in DNA derived from tumor tissues and ctDNA. In patients with paired tissue and blood samples, a total of 206 SNVs and 43 short Indels were detected (Figure 2B). The frequency of mutations and the consistency between tissue and blood samples are shown in Figure 2B. The most consistent gene was *TP53* (81%), followed by *APC* (67%) and *KRAS* (42%).

The consistency at individual CRC stages was determined by comparing the detection of mutations in ctDNA and corresponding tumor tissues for each patient (Figure 2C). It can be observed that the consistency for stage IV patients distributed in a wide range, and 24% of patients exhibited a consistency >0.75 (7/29), while 76% of patients exhibited a consistency <0.75 (22/29). The tTMB (tissue TMB) between those with a consistency >0.75 and those <0.75 was not statistically different (Figure 2D), although the trend showed that the group with lower consistency (<0.75) had a lower tTMB.

### Treatment Significantly Altered Mutational Landscape in ctDNA

A NGS panel containing 605 genes was used to monitor the genetic alterations following the therapy. Here we compared the alterations of gene mutations at different time points following treatment to assess the potential effect of therapy and to find any potential instruction on therapeutic strategy. Four specific cases were presented to illustrate the significance of NGS assay in clinical treatment.

Patient 00601 first presented adenocarcinoma (stage IV), and bilateral lung metastasis were found 6 months after surgery. The patient was treated with Bevacizumab and Nivolumab, and blood samples were collected 1 year after treatment. *BRAF* V600E and *SMAD4* R361H were still detected after a series of therapies compared with previously surgical resected samples. Although mutations in some genes (such as *CCNE1, DICER1, MSH2*, and *PIK3CD*) were altered, new mutations (*ATM* p.L2541P, *ATM* p.V2540L, and *NF1* p.P2742L) were identified (Figure 3). We speculate that these new mutations, combined with those that still existed, may suggest the development of drug resistance or metastasis.

Patient 00603 presented sigmoid adenocarcinoma (T4aN0M1), and metastasis was found in the liver, lung, and lymph nodes. A combined treatment of Bevacizumab and Apatinib with FOLFIRI was applied but did not substantially alter the key driver gene mutations in *APC, ASXL1, BARD1*, and *KRAS* (Figure 3). These mutations could correlate with disease progression and poor overall prognosis of the patient, as he only survived 194 days after confirmation of diagnosis.

Patient 00606 presented rectal cancer (stage IV) with lung and brain metastasis. Blood samples were collected after 1 week treatment with Bevacizumab and FOLFIRI (second-line treatment). However, gene mutations of *APC, ERBB2, IKZF1, KRAS, and RAF1* were still detected after treatment compared with pre-therapeutic results. Moreover, new mutations of *APC, IRS2, NR4A3, NTRK1, PRDM1*, and *VEGFA* were detected. Discover of these new mutation normally suggest disease progression or new metastasis, which was proved by the clinical status of PD of the patient (Figure 3).

Patient 00608 presented rectal cancer (stage IV) and lung metastasis. Blood sampling was also performed to investigate the disease progression following Bevacizumab and FOLFOX6 treatment. The genetic variation from needle biopsy samples of pulmonary metastases greatly differed from that of the blood detection (Figure 3). Novel *RNF43* mutation sites in ctDNA were identified, supporting previous observation showing that *RNF43* frameshift mutation may contribute to tumorigenesis (6).

### Comparison of Key Mutations in Baseline and After First-Line Therapy

We monitored key mutation changes of 13 patients between baseline and after the first-line therapy of bevacizumab, cetuximab with chemotherapy and grouped the patients by response [progressed diseases (PD), partial response (PR), and stable disease (SD)]. The key mutations were different between baseline tissues and progressive ctDNA samples in all three groups (Figure 4). Most of the genes were reported to contribute to the carcinogenesis of CRC. Compared with baseline, a new mutation (*PDGFRB* p.Q443R) was observed during disease progression. More importantly, the combination of bevacizumab/chemotherapy or cetuximab/chemotherapy was able to alleviate the mutation of the driving gene, such as *BRAF, KRAS, AMER1*. Mutations in *PPIAL4D* p.S99F and *SPATA31A5* p.T1139R were not observed after therapy. The above observations suggest that mutational profile of ctDNA may help to determine the response of patients to treatment.

### *TP53*/*KRAS* Mutations in Tumor Tissue Are Potential Predictive Factors for Treatment Response

In this study, 18 patients were treated with bevacizumab combined with chemotherapy, while 4 patients were treated with cetuximab combined with chemotherapy, and 2 with chemotherapy alone. We divided these patients into two groups by *TP53/KRAS* co-mutation. It can be seen from the survival analysis in Figure 5 that PFS appeared to be shorter for patients with no *TP53/KRAS* co-mutation, with a median PFS of 381 days for no co-mutation group compared with 460 days in *TP53/KRAS* co-mutation group (*p* = 0.13). Patients with *KRAS* and *TP53* co-mutations in tissues exhibited a potentially better response to treatment than those containing the respective wild type genes. The relationship between the prognosis of first-line therapy and the *TP53/KRAS* co-mutations is worth more investigation.

## Discussion

In this study, we explored the practicability and clinical value of ctDNA in CRC therapy using paired blood and tissue biopsy samples. To clarify the correlation between drug efficacy and mutations, we built up mutational profiles for 43 CRC patients. We dynamically monitored the mutation status of each patient and investigated its relationship with therapeutic response, including drug resistance (10). Our study revealed many gene mutations with the 605-gene panel. Apart from previously reported mutations in *APC, TP53, KRAS, SYNE1, PI3KCA, SMAD4*, and *BRAF*, some other mutations may also potentially be used as biomarkers for CRC prognosis. We also showed that ctDNA may be used to analyze TMB, which is an effective method to monitor the burden of mutations following therapy. The consistency between ctDNA and tissue biopsy was 32%.

We collected plasma ctDNA and tumor tissues in CRC patients following cetuximab, apatinib, trastuzumab, neoadjuvant, adjuvant chemotherapy, or any combination of them to track tumor dynamics, including therapeutic response, metastasis, and drug resistance. Despite the limited cohort (*n* = 41), the samples represented patients of advanced CRC with integrated clinical information. Four representative patients with treatment plans and mutational changes were presented and analyzed in detail (Figure 3). Although the mutational profile of each patient was distinct, our results indicated that the mutation of driver genes dynamically changed in different patients and treatment plans. Therefore, it is vital to detect these gene mutations in an individualized manner. We believe this strategy can support the establishment of reasonable treatment plan for each patient (11–14).

After comparing key mutation profiles between baseline and after first-line therapy (Figure 4), a new mutation (*PDGFRB* p.Q443R) was observed during disease progression. Consistent with previously observations, *PDGFRB* appeared to promote the development of CRC (15, 16). Moreover, combinations including bevacizumab-chemotherapy and cetuximab-chemotherapy could diminish the mutations of driving genes, such as *BRAF, KRAS, AMER1*, and therefore potentially prevent disease progression. *BRAF* and *KRAS* are driver genes of CRC, while *AMER1* is a frequently mutated gene in this condition (17). Mutations in two other genes (*PPIAL4D* p.S99F and *SPATA31A5* p.T1139R) were previously rarely reported, which may constitute alternate mutation sites that deserve more in-depth investigation. Since gene mutations typically accumulate over time, CRC with distinct heterogeneity may exhibit different genetic characteristics. The mutational information from a single tissue biopsy is limited by space and time, and may be biased in accessing the therapeutic effect or monitoring cancer progression. Ideally, multiple biopsies may be obtained to avoid this bias. Although we did not achieve ideal condition, our study improved the understanding in the roles of ctDNA in CRC monitoring and response assessment in the practice of precision medicine.

*TP53* mutations in early CRC have been considered a poor prognostic factor. However, the function of mutated TP53 has not been fully characterized (18). In our study, *TP53* and *KRAS* mutations were considered favorable factors for overall survival and disease progression of CRC. PFS was potentially shorter for patients without *TP53/KRAS* co-mutations compared with *TP53/KRAS* mutated patients and therefore might have more treatment benefit (Figure 5). Indeed, these two genes were often found co-mutated in our sequencing results. Therefore, although some potential interesting findings were revealed in the current study, it requires further validation using a larger number of patients. In this study, we confirmed the roles of ctDNA in dynamic monitoring of CRC therapy, which supports more extensive use of the method in future therapy.

In this study, we monitored the mutational changes and therapeutic response of late-stage CRC patients following chemotherapy combined with bevacizumab and/or cetuximab. It appeared that high frequency mutations of key driver gene, such as APC, TP53, and KRAS, were the markers that sensitively reflected the therapeutic response, while alleviation of mutations in BRAF, AMER1, and other major driver genes was also observed following the therapy. The dynamic change of plasma mutation status following the combined therapy was consistent with the tissue tumor burden and was closely correlated with disease progression. Therefore, ctDNA detection appeared to be a useful method for the molecular genotyping and sensitive for monitoring mutational status, which potentially helps to develop treatment strategy targeting actionable variations. Our observations were supported by several previous reports focusing on the monitoring capability of NGS-based liquid biopsy in late-stage CRC therapy (4, 19–24). However, the regimes used in these studies varied according to different situation. Some studies focused on the monitoring of cetuximab-based therapy for RAS wild type patients (19–21), while others emphasize the monitoring of multiple targets, including EGFR, HER2, SMAD4, and NF1 (4, 21–23). Interestingly, one study investigated the ability of both mutation and methylation markers in monitoring (24). Although the therapeutic regimes and targets varied, the observations from these studies all support the use of NGS-based liquid biopsy for therapeutic response monitoring and target identification, which endorsed our conclusions.

There were some limitations in this study. Firstly, the sample size was still small. Although 43 patients were included, only 35 patients had paired baseline plasma and corresponding tumor tissues, and key mutation changes between baseline and after the first-line therapy were obtained from 13 patients. Therefore, incomplete paired samples and loss of follow-up were key issues in the study, which increased the difficulties in analysis and making solid conclusion. These issues can be solved by increasing the number of total subjects and patients with complete information may increase accordingly. Meanwhile, strict fulfillment of inclusion and exclusion criteria may also increase the ratio of patients with complete information. Secondly, the therapeutic strategy in this study varied among different patients. Therefore, studies on mutational changes may be differentially affected by various chemotherapy drugs, and conclusions based on mixed therapies may be compromised. It would be nice to study the mutational changes with patients from homogeneous treatment, however, this was difficult operationally, as late-stage CRC patients from multiple lines of therapy generally have diversified conditions and will adopt different therapies in the real world.

## Data Availability Statement

The datasets generated for this study can be found in the Genome Sequence Archive for Human (GSA-Human) (Accession: PRJCA002282).

## Ethics Statement

The studies involving human participants were reviewed and approved by Shenzhen People's Hospital. The patients/participants provided their written informed consent to participate in this study.

## Author Contributions

RX designed the study. HC and XL performed the sample collection, data collection, and manuscript writing. YC, PY, and TH performed the sequencing and data analysis. XL, PY, and LS wrote the manuscript. LS and RX proof read the manuscript.

### Conflict of Interest

XL, PY, TH, and LS are currently employed by HaploX Biotechnology. HaploX provided the next generation sequencing service for this study. The remaining authors declare that the research was conducted in the absence of any commercial or financial relationships that could be construed as a potential conflict of interest.
